# Small and large fiber neuropathy in adults with myotonic dystrophy type 1

**DOI:** 10.3389/fneur.2024.1375218

**Published:** 2024-03-05

**Authors:** Gro Solbakken, Sissel Løseth, Jan C. Frich, Espen Dietrichs, Kristin Ørstavik

**Affiliations:** ^1^Institute of Clinical Medicine, University of Oslo, Oslo, Norway; ^2^Department of Neurology, Rheumatology and Rehabilitation, Drammen Hospital, Vestre Viken Health Trust, Drammen, Norway; ^3^Department of Clinical Medicine, The Arctic University of Norway, Tromsø, Norway; ^4^Section of Clinical Neurophysiology, University Hospital of North Norway, Tromsø, Norway; ^5^Institute of Health and Society, University of Oslo, Oslo, Norway; ^6^Department of Neurology, Oslo University Hospital, Oslo, Norway

**Keywords:** myotonic dystrophy 1, small-fiber, skin biopsy, large fiber, neuropathy, CTG size, pain

## Abstract

**Introduction:**

Myotonic dystrophy type 1 (DM1) is an inherited neuromuscular disorder that affects multiple organs. In this study, we investigated symptoms of pain and presence of small and large fiber neuropathy in the juvenile and adult form of DM1.

**Method:**

Twenty genetically verified DM1 patients were included. Pain was assessed, and neurological examination and investigations of the peripheral nervous system by quantification of small nerve fibers in skin biopsy, quantitative sensory testing and nerve conduction studies were performed. Results from skin biopsies were compared to healthy controls.

**Result:**

Seventeen patients reported chronic pain. Large and/or small fiber abnormalities were present in 50% of the patients. The intraepidermal nerve fiber density was significantly lower in the whole group of patients compared to healthy controls.

**Conclusion:**

Small-fiber neuropathy might be an important cause of pain in DM1.

## 1 Introduction

Myotonic dystrophy type 1 (DM1) is the most frequent hereditary muscle disease in adults (1). DM1 does not only impair muscle strength, but also involves multiple organs. Some studies have shown that DM1 may involve the peripheral nervous system (2–4). Recently Boland-Freitas and Ng found *indications* of a subclinical small fiber neuropathy measured by quantitative sensory testing in patients with DM1 (5). Chronic pain has been reported in about 60%, and has been associated with fatigue, reduced motor function and quality of life (6–8). Pain location is widespread, and pain in hands and feet is frequent (8). This pain distribution, together with previous studies on large and small fiber neuropathy, could indicate that there might be a neuropathic component of pain in DM1. Pathology of small nerve fibers (C fibers and Aꝺ-fibers) is essential for peripheral neuropathic pain (9). These fibers may be tested by the semi-objective method of quantitative sensory testing (QST), while the gold standard is skin biopsy for quantification of intraepidermal nerve fiber density (IENFD) (9). Indeed, a recent review on diagnosis of neuropathic pain using the GRADE system found that skin biopsy is so far the *only* investigation to confirm peripheral neuropathic pain (10). To our knowledge, this has not previously been performed in patients with DM1. In this cross-sectional study, we investigated the presence of pain and large and small fiber neuropathy including IENFD in a group of patients with the juvenile and adult forms of DM1.

## 2 Materials and methods

### 2.1 Recruitment and inclusion

DM1-patients with the classic form (juvenile and adult) from the North and South-Eastern regions of Norway, were invited to participate in a larger clinical study on DM1 as previously reported (11, 12). Patients were contacted through the National Registry of Neuromuscular Disorders, via the patient organization’s journal, and through hospitals. Thirty-two patients were invited to participate in this sub-study on neuropathy and pain. Patient invitations were limited to those living within a 2 h drive to the hospitals where the study was performed. Exclusion criteria were diagnosed and treated diabetes, hypothyroidism, autoimmune disorders, and a known diagnosis of peripheral neuropathy, cancer, or the use of medication that may cause neuropathy or cognitive decline. In addition, the most severely impaired patients (e.g., patients who needed support from another person to be able to travel to the investigation site) were excluded. A total of 20 out of 32 patients accepted the invitation to participate.

Disease duration was calculated based on time between onset of DM1 symptoms and inclusion in the present study (13). Southern blot analysis for number of CTG size (1) was obtained from all patients.

#### 2.1.1 Clinical measures

All patients went through a general neurological examination of the peripheral nervous system including sensory testing for light touch and pinprick in the lower extremities, and deep tendon reflexes.

Muscle strength were assessed by manual muscle strength test (MMT) and scored according to the Medical Research Council’s (MRC) 0–5 categories (14). Only one side was tested (15). MMT results were categorized into the DM1 specific muscular impairment rating scale 1–5 (MIRS grade 1: no clinical signs, grade 2: clinical myotonia, minimal signs of muscles involved in the face and neck, grade 3: muscle impairments in the distal part of the extremities, grade 4: proximal muscle involvement in addition to distal muscles, grade 5: severe proximal involvement) (16).

We used pain drawings to collect data about presence of pain (17). A Numeric Rating Scale (NRS) from 1–10; 0: no pain, mild scores: 1–3, moderate scores: 4–6, severe scores: 7–10, was used for registration of pain intensity (18). Patients were instructed to mark mean pain intensity of chronic pain (pain lasting more than 3 months). Further, they were asked to describe the quality of pain sensations like burning, lancinating or deep pain.

#### 2.1.2 Neuropathy measurements

Large fibers were investigated with nerve conduction studies (NCS), and small fibers with QST of thermal thresholds and skin biopsy for quantification of IENFD. NCS were performed on Keypoint Classic^®^ and Keypoint G4^®^ machines. Motor and sensory nerve conduction velocities (NCV) and amplitudes of the median and ulnar nerves in one upper extremity were examined. In the lower extremities the motor NCV, amplitudes of the peroneal and tibial nerves as well as the sensory amplitudes and NCV of the sural, medial plantar and peroneal superficial nerves were examined in both legs. The NCS results were compared with normal values in use in the laboratories. In this study, we regarded pathological findings in three nerves in the lower extremities including at least one sensory nerve as compatible with large fiber neuropathy. Heat detection thresholds (HDT), cold detection thresholds (CDT) and heat pain detection thresholds (HPDT) were determined using a computerized Thermotest^®^ (Somedic AB, Sweden) as described elsewhere (19). HDT and CDT were calculated as the average of five consecutive temperature recordings. These thresholds were determined at the thenar eminence of the left hand, at the lateral aspect of the left thigh, at the lateral aspect of the left leg approximately 15 cm below knee level, and at the dorsum of the left foot. HPDT was determined at the dorsum of the foot and calculated as the average of three recordings at 10 s intervals. Thresholds were compared to normal material obtained in our lab (20). Findings of increased thresholds for either CDT, HDT or both at the dorsum of the foot indicated small fiber neuropathy.

Two skin biopsies were obtained from the distal part of the leg, 5–10 cm above the lateral malleolus, with a 3-mm disposable circular needle under local anesthesia. Fifty-micron freezing sections were immunostained with the panaxonal marker PGP 9.5. The number of separate intraepidermal nerve fibers (IENFs) in three sections from each biopsy was counted, and the total length of epidermis was measured. IENFD was then calculated as the mean of counts in these six sections. IENFD in patients was compared with data from 106 healthy adult individuals analyzed in the same laboratory (21).

#### 2.1.3 Statistics

The SPSS 25 (IBM Corporation Armonk, NY, United States) was used for calculations. The distribution of the variables is presented with mean, standard deviation (SD), range and median. IENFD *Z*-scores were calculated from the reference material (22) after log transformations, taking age and gender into account. IENFD was defined as abnormal in a patient if *Z*-score was ≤ −2.0. IENFD (both absolute values and *Z*-scores) in DM1 patients were compared to the reference group with student’s *t*-test. Effect sizes (Cohens *d*) were calculated using the online social science statistics service: http://www.soscistatistics.com/effectsize/Default3.aspx. Cohens *d* at 0.2 were interpreted as small, 0.5 as medium and >0.8 as large. *p*-values were set at two tailed <0.05.

## 3 Results

Twenty patients participated in the study (Table 1). Participants were mildly to severely affected according to CTG size, strength measures and disease duration. These measures did not differ between men and women.

**Table 1:** Characteristics of the 20 study participants with the juvenile and adult form of DM1.

Measures	Mean, SD, (range) [median] Number (*N*)
Age, years	38.8, SD: 12.8, (19–62) [40.5]
Gender	13 women, 7 men
CTG kb	1.8, SD: 1.3, (0.270–4.5) [1.5]
Disease duration, years	18.6, SD: 9.7, (5–39) [19.5]
IQ (*n* = 16)	96, SD: 12, (74–115) [96]
Mean strength	4.0, SD: 0.6, (3–5) [4]
MIRS	2.8, SD: 1.1, (1–5) [2.5]
Pain intensity (NRS)	5.5, SD: 2.4, (0–8) [6]
IENFD depletion	2 (*N*)
NCS abnormal	6 (*N*)
QST abnormal	4 (*N*)

CTG, cyanine, thymine, guanine nucleotide; MIRS, muscular impairment rating scale; NRS, numeric rating scale; IENFD, intraepidermal nerve fiber density; NCS, nerve conduction studies; QST, quantitative sensory testing.

Number of participants in MIRS category 1–3 (mild to moderate) was 14, and in MIRS category 4–5 (severe) was 6. The CTG repeat size was distributed as follows: very small (50–100 CTG repeats) in 1 patient, small (101–200 CTG repeats) in 2 patients, medium (201–700 CTG repeats) in 10 patients and large (>700 CTG repeats) in 6 patients.

### 3.1 Symptoms of pain

Seventeen patients (85%) reported chronic pain. Of these, six patients reported symptoms possibly indicating neuropathic pain, like burning or lancinating pain in their feet. Fourteen patients reported other types of pain qualities (deep, aching muscle pain and musculoskeletal pain), and three reported both types of pain.

### 3.2 Clinical examination

We found decreased or absent deep tendon reflexes in at least two sites in the lower extremities in 14 patients. Six patients had decreased distal sensibility for pinprick or light touch or both.

### 3.3 Objective and semi-objective findings of large or small fiber neuropathy

**Figure 1:**
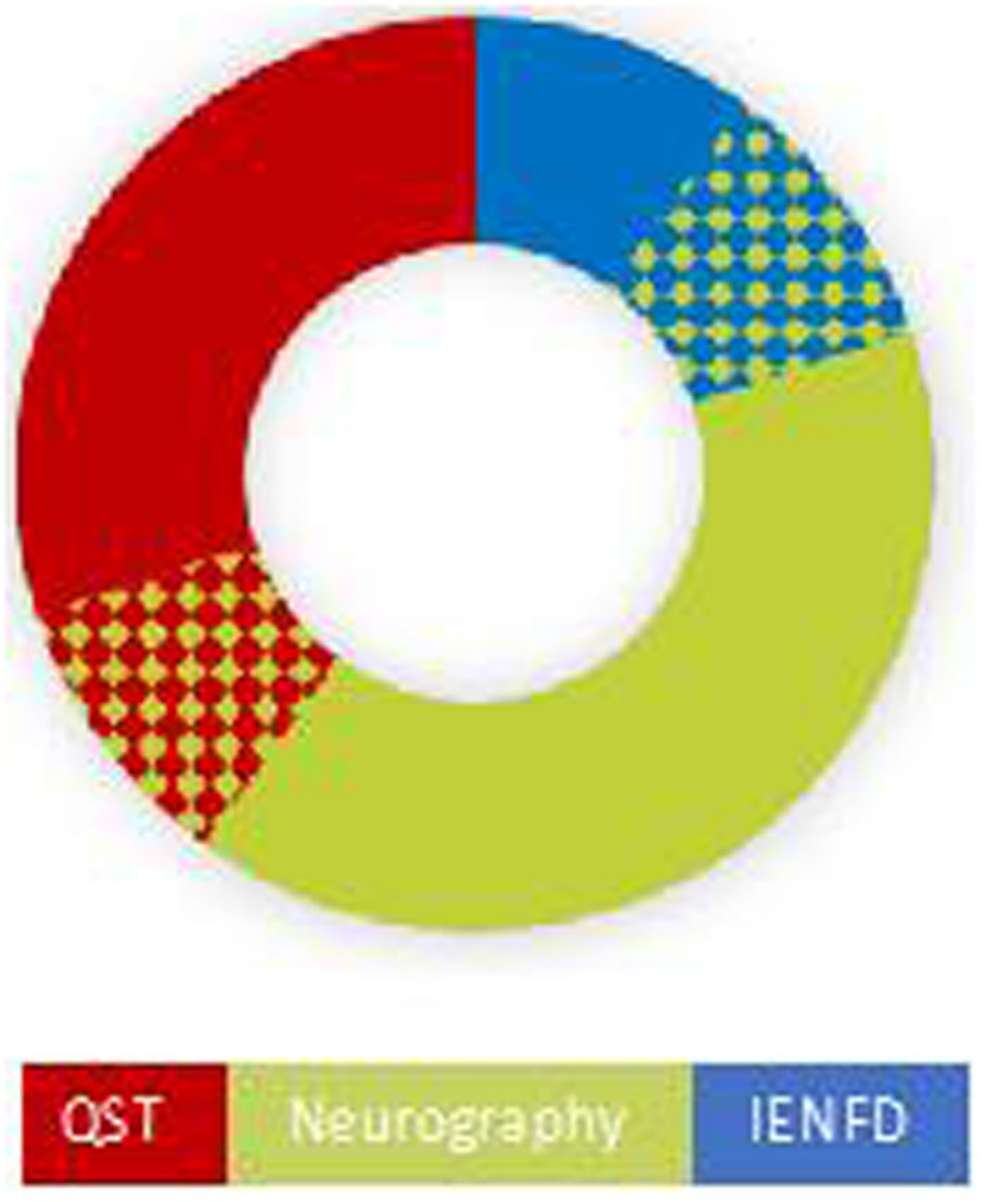
Distribution of objective (Neurography and IENFD) and semiobjective (QST) findings of neuropathy in 10 patients. Six had findings compatible with large fiber neuropathy, four had abnormal findings on QST, and two had abnormal skin biopsy (IENFD). There was an overlap between large fiber findings and the two small fiber tests, as shown by the chess pattern.

In 10 patients NCS and/or QST and/or IENFD revealed abnormal findings (Table 1 and Figure 1).

Only two patients (females aged 37 and 40) had a mild depletion of small fibers in skin biopsy (4.5 fibers/mm and 5.2 fibers/mm; normal values calculated from our reference material taking their age and gender into account is >6.6 fibers/mm). These two did not have symptoms typical for neuropathic pain. However, when comparing absolute values of IENFD in the DM1 group (*N* = 20, mean 8.2 fibers/mm, SD: 2.28) to the reference group (*N* = 106, mean 12.4 fibers/mm, SD: 4.59), there was a significant difference (*p* ≤ 0.001, Cohen’s *d* = 1.2) with a lower number of nerve endings in the patient group compared to controls. This difference was also significant when using IENFD *Z*-scores (DM1 group mean −1.16, SD: 0.8. Reference group: mean 0.05, SD: 1.0, *p* ≤ 0.001, Cohen’s *d* = 1.8).

Of the six patients reporting pain distally in their feet, two patients had pathological QST findings, while one patient had abnormal NCS. These three patients also had positive clinical sensory findings with hyperalgesia for pin-prick in the painful area.

### 3.4 Neuropathy findings and DM1 symptoms

Patients with large fiber neuropathy as measured by NCS (*n* = 6) had significantly lower mean muscle strength (*p* = 0.009) than patients without large fiber neuropathy. In addition, the number of CTG repeats showed a tendency to be higher in the group with large fiber neuropathy. However, this difference did not reach significance in this small group of patients (*p* = 0.072). All the patients with abnormal NCS findings had absent reflexes. Patients with absent reflexes (n = 14) had significantly lower muscle strength (*p* = 0.001) compared to the patients with normal reflexes.

## 4 Discussion

In this study of 20 adult patients with DM1, 17 patients reported symptoms of pain, six of which suggestive of a neuropathic type. Ten patients had abnormal findings on NCS, skin biopsy or QST or a combination of them. IENFD was significantly lower in the DM1 group compared to healthy controls.

In the present study, large fiber neuropathy documented by NCS was related to reduced muscle strength and is in line with previous studies on peripheral neuropathy in DM1 patients (2). Patients with pathological NCS had larger CTG size, and the same has been found in a DM1 mouse model (23). Neuropathy in patients with DM1 may therefore be associated with more severe disease, which usually correlates to CTG size.

We investigated the presence of small-fiber neuropathy by both QST and skin biopsies. However, QST is a semi-objective method, and there is a risk both of false positive and false negative findings (24). Quantification of IENFs in skin biopsy is considered to be the gold standard for the diagnosis of small fiber neuropathy in symptomatic patients (25). Two patients in our study had depletion of IENFs, one of them had also abnormal NCS consistent with a mixed small- and large fiber neuropathy (Figure 1). None of these patients reported neuropathic pain. This may be due to the fact that neuropathy (small and large fiber) do not necessarily lead to neuropathic pain (26). None of our patients had definite small fiber neuropathy according to Besta and Neurodiab criteria (27, 28). Besta criteria require two objective clinical signs and abnormal QST or IENF. Neurodiab criteria require the presence of a single clinical sign, normal sural nerve conduction study and either abnormal QST or IENFD.

An interesting finding is the difference of IENFD between the DM1 group and healthy subjects, which may indicate that subclinical small fiber involvement may be present in DM1 patients (21, 22). Patients with DM1 have a higher risk of developing diabetes. However, none of the patients included in the present study had a diagnosis of diabetes. Diabetes is thus not a plausible cause of the presence of small and large fiber neuropathy. Furthermore, none of them had been diagnosed with cancer, so paraneoplastic neuropathy was considered very unlikely among our patients. Patients with DM1 may have central nervous system involvement that could play a role in the development of neuropathic pain (26). However, this does not explain the findings on skin biopsies. Meinke et al. (29) suggest that DM1 may be a progeroid syndrome. This could, be a possible explanation for our findings of small-fiber neuropathy.

Seventy percent of patients in our study reported other pain qualities than neuropathic pain. A high frequency of pain, located in both the extremities and the trunk, has previously been documented in DM1 patients (30). A possible explanation for chronic pain in DM1 patients could be a consequence of increased and unbalanced weight on joints and ligaments because of myopathy, as well as the myopathy itself resulting in muscle tissue damage that could cause nociceptive pain (31). Another well-known symptom in DM1 patients is myotonia or muscle cramps. This phenomenon varies between patients, for some it may be disabling and painful, for others less bothersome (32).

### 4.1 Strengths and limitations

The main strength of our study is the combination of subjective and objective measurements of neuropathy, including skin-biopsies, which represents a gold standard for the diagnoses of small fiber neuropathy (9, 27). To our knowledge, this is the first study using skin biopsy to evaluate the presence of small fiber neuropathy in DM1 patients. Another strength of our study is the well-defined patient group with genetic verification of the diagnosis for all participants. A cross-sectional design does not allow us to study changes over time. Further, the sample size is small and can only capture large effect sizes, smaller effects are likely to be missed. Another limitation is that we did not use standardized questionnaires for neuropathic pain. A recently published guideline on assessment of neuropathic pain using the GRADE system showed that only three of the many questionnaires in use are highly recommended, while IENFD is the only diagnostic method that reaches the same level (10). Other possible symptoms and findings of small fiber neuropathy such as change of swelling, orthostatism and other autonomic signs were not explored in this study. Future studies on DM1 and pain should include more subjects as well as one of these recommended questionnaires.

## 5 Conclusion

We document the presence of both large and small fiber neuropathy in DM1 patients. A high proportion of patients reported chronic pain. IENFD was lower in the DM1 group compared to healthy controls. We conclude that small and large fiber neuropathy may be a mechanism of pain in DM1.

## Data availability statement

The raw data supporting the conclusions of this article will be made available by the authors, without undue reservation.

## Ethics statement

The studies involving humans were approved by the Regional Committees for Medical and Health Research Ethics, South East, Norway. The studies were conducted in accordance with the local legislation and institutional requirements. The participants provided their written informed consent to participate in this study.

## Author contributions

GS: Writing – original draft, Writing – review & editing. SL: Conceptualization, Data curation, Formal analysis, Funding acquisition, Investigation, Methodology, Project administration, Resources, Software, Supervision, Validation, Visualization, Writing – review & editing. JF: Conceptualization, Data curation, Formal analysis, Funding acquisition, Investigation, Methodology, Project administration, Resources, Software, Supervision, Validation, Visualization, Writing – review & editing. ED: Conceptualization, Data curation, Formal analysis, Funding acquisition, Investigation, Methodology, Project administration, Resources, Software, Supervision, Validation, Visualization, Writing – review & editing. KØ: Conceptualization, Data curation, Formal analysis, Funding acquisition, Investigation, Methodology, Project administration, Resources, Software, Supervision, Validation, Visualization, Writing – review & editing.
